# A Case of Paracentral Corneal Perforation Treated with One-Bite Mini-Keratoplasty

**DOI:** 10.4274/tjo.galenos.2020.40111

**Published:** 2021-02-25

**Authors:** Yoshitake Kato, Daisuke Nagasato, Shunsuke Nakakura, Taiichiro Chikama, Chikako Katakami, Hitoshi Tabuchi, Yoshiaki Kiuchi

**Affiliations:** 1 Saneikai Tsukazaki Hospital, Department of Ophthalmology, Himeji, Japan; 2 Hiroshima University Graduate School, Department of Technology and Design Thinking for Medicine, Hiroshima, Japan; 3 Graduate School of Biomedical Sciences, Department of Ophthalmology and Visual Sciences, Hiroshima University, Japan

**Keywords:** Aqueous humor leakage, corneal perforation, one-bite mini-keratoplasty, single suturing

## Abstract

A 61-year-old man presented with corneal perforation of 1.0 mm in diameter in his right eye caused by a metallic foreign body fragment. We used the “one-bite mini-keratoplasty” technique, which uses a cornea patch with a single host-graft-host suture to stop aqueous humor leakage. Postoperatively, the graft was completely epithelialized. The suture was removed and the use of soft contact lens was discontinued. Postoperative best-corrected visual acuity (BCVA) recovered to 180/200 and corneal astigmatism was 0.6 diopters. The postoperative course was unremarkable, but corneal perforation recurred due to an ocular contusion at 17 months. He was reoperated using the same technique. His BCVA was 160/200 and corneal astigmatism was 1.1 diopters after reoperation. Despite performing this surgical technique twice for corneal perforation, optimal visual function was maintained even after 2 years. For paracentral corneal perforations, our simple technique may reduce astigmatism and maintain high visual function.

## Introduction

Corneal perforation is an emergency condition that may occur due to eye trauma or infectious corneal disorders and leads to hypotony. Although it is necessary to close the perforation, strong astigmatism is likely to occur with simple sutures and conventional lamellar keratoplasty for paracentral corneal perforation. To improve visual acuity and prevent corneal irregular astigmatism as much as possible, we performed “one-bite mini-keratoplasty,” in which insertion of a very small corneal graft into the paracentral corneal perforation was achieved using a single suture to the host cornea. Using this new approach, we successfully treated the patient’s corneal perforation and also minimized astigmatism.

## Case Report

**Figure 1:**
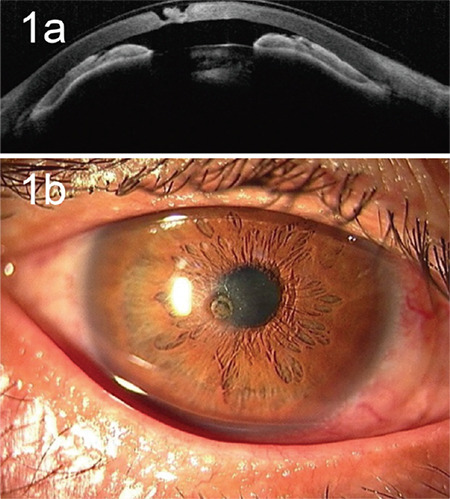
Preoperative images of the paracentral corneal perforation. a) The anterior chamber was not formed even with the use of a soft contact lens, as visualized using anterior segment optical coherence tomography. b) A corneal defect approximately 1.0 mm in diameter was recognized near the center of the cornea via slit-lamp photography

A 61-year-old man consulted an ophthalmologist with a foreign body in his right eye. Corneal foreign body was detected. During the removal of the foreign body, corneal perforation was observed. Thus, he consulted our hospital with a soft contact lens (SCL). At the initial visit, the best-corrected visual acuity (BCVA) in his right eye was 20/200. Slit-lamp examination and anterior segment optical coherence tomography revealed a flat anterior chamber (Figure 1a) and a paracentral corneal perforation approximately 1.0 mm in diameter spanning a small portion of the deep stroma and endothelium (Figure 1b). Upon removal of the SCL, aqueous humor leakage was confirmed with a positive result for Seidel test using fluorescein. Although the SCL mitigated the condition for 3 weeks, the leakage did not stop; therefore, we elected to perform one-bite mini-keratoplasty in the right eye.

**Figure 2:**
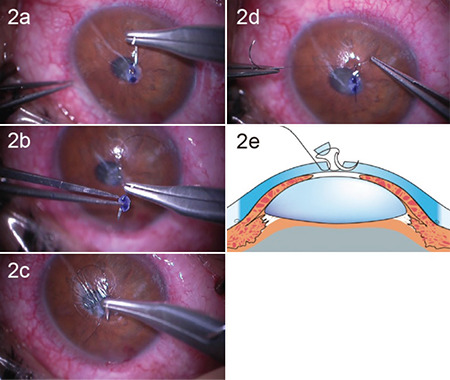
Surgical technique of “one-bite mini-keratoplasty.” a) Step 1: passing the needle through the host cornea; b) Step 2: passing the needle through the prepared graft, c) Step 3: passing the needle through the host cornea on the opposite side, d) Step 4: Tying the suture; e) Schematic drawing of one-bite mini-keratoplasty

After sub-Tenon’s anesthesia of the right eye with 2% lidocaine, we manually excised a portion of the endothelial stroma of a preserved cornea and prepared a graft of approximately the same size as the corneal perforation. Without inserting viscoelastic material into the anterior chamber, we passed a single 10-0 nylon suture (horn needle, part number 1404, MANI) through the host cornea (Figure 2a), then through the prepared graft (Figure 2b) and into the host cornea on the opposite side (Figure 2c), and tied the suture (Figure 2d). This schema is shown in Figure 2e. The graft was fitted into the perforation. After confirming that there was no aqueous humor leakage, an SCL (Acuvue Oasys, Johnson&Johnson, Jacksonville, USA) was placed and the surgery was completed.

**Figure 3:**
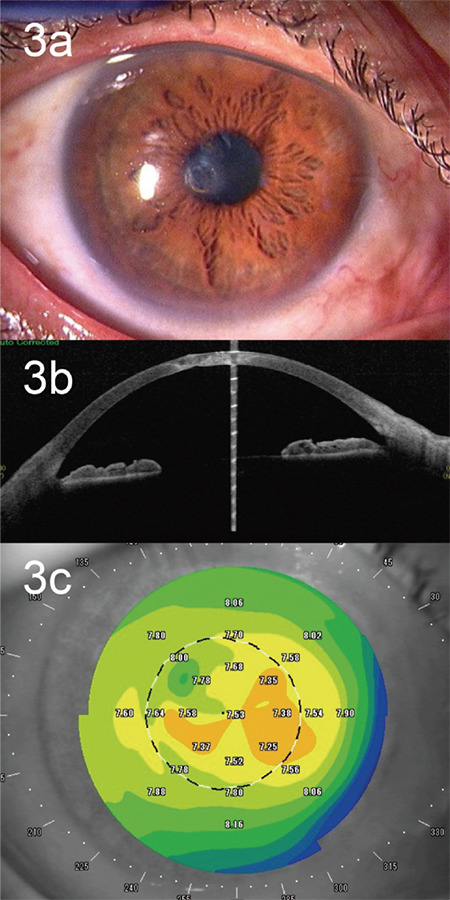
Postoperative images of paracentral corneal perforation. a) The corneal defect was plugged with a corneal graft, and the graft epithelialized; b) Anterior segment optical coherence tomography showed the corneal graft filling the corneal defect with a well-formed anterior chamber; c) The cylinder value was 1.1 diopters, and the astigmatism was mild as visualized using corneal topography

The following day, the anterior chamber had reformed and Seidel test result was negative. Postoperatively, the patient was treated with 0.1% fluorometholone and 1.5% levofloxacin 4 times daily. One month postoperatively, the graft was completely epithelialized. Two months postoperatively, we removed the suture and discontinued use of the SCL. Postoperative BCVA recovered to 180/200, and corneal astigmatism was 0.6 diopters. However, at 17 months, corneal perforation recurred because of ocular trauma, and a flat anterior chamber and partial laceration at the graft-host junction were observed. We attempted to reform the anterior chamber using an SCL but were unsuccessful. Because the original graft was damaged, the patient underwent a repeat one-bite mini-keratoplasty with a new graft. The postoperative course was similar to that of his first surgery, and the graft was completely epithelialized 2 weeks postoperatively. We removed the suture and discontinued the use of the SCL 4 months postoperatively. Subsequently, graft adhesion was good (Figure 3a) and the epithelium remained stable even approximately 2 years postoperatively (Figure 3b). BCVA was 160/200 and corneal astigmatism was 1.1 diopters (Figure 3c).

## Discussion

Use of SCL is an effective approach to suppress aqueous humor leakage occurring as a consequence of corneal perforation.^1^ However, large corneal perforations cannot be prevented using SCL; furthermore, simple suturing causes visual function deterioration due to strong corneal irregular astigmatism. Amniotic ^2^ and fibrin glue-assisted amniotic membrane transplant methods ^3,4^ have been reported to alleviate corneal astigmatism, but these procedures are more complex than our technique. Lamellar keratoplasty is another procedure that is effective against perforation.^5^ However, this procedure is associated with a possibility of negatively affecting visual function when using a graft larger than the perforated area in cases where the perforation is near the center of the cornea. We performed one-bite mini-keratoplasty in a patient in whom aqueous humor leakage could not be stopped using an SCL. Because the diameter of the corneal defect was 1.0 mm, we initially considered conventional lamellar keratoplasty; however, due to the difficulty of applying several sutures radially around the graft, we adopted the single-suture method using as a filler a corneal graft of approximately the same size as that of the corneal defect. Previous studies have reported the use of “small-diameter keratoplasty”^6,7^ or “small-diameter graft,”^7,8^ but the use of a small-diameter graft approximately 1.0 mm in size or a suturing method with a single suture has not been previously reported. Chern et al.^6^ reported severe irregular astigmatism after a “small-diameter, eccentric penetrating keratoplasty” was performed for paracentral corneal perforation. In our patient, the first surgery using this technique showed successful results with little astigmatism. Also, we observed corneal astigmatism of 1.1 diopters and BCVA of 160/200 despite the paracentral corneal re-perforation secondary to ocular trauma that occurred after performing one-bite mini-keratoplasty.

Corneal tissue is sturdier than amniotic material, and suturing tangentially to the cornea can help minimize corneal astigmatism. In addition, because corneal grafts can be secured without gaps, our technique enables plugging the aqueous humor leakage more reliably than using an amniotic membrane. The corneal tissue provides a scaffold for promoting corneal epithelialization in addition to filling the defect site. In this case, the graft epithelialized and remained stable over the long term. Therefore, in our patient, one-bite mini-keratoplasty resulted in the maintenance of high visual function by suppressing aqueous humor leakage, helping attain corneal epithelialization, and maintaining good visual acuity. This technique is useful in settings where donor cornea is available. Therefore, one-bite mini-keratoplasty could help preserve long-term corneal stability and good visual function including minimize corneal astigmatism for the treatment of paracentral corneal perforation.
